# A longitudinal study of the association between domestic contact with livestock and contamination of household point-of-use stored drinking water in rural Siaya County (Kenya)

**DOI:** 10.1016/j.ijheh.2020.113602

**Published:** 2020-09

**Authors:** Diogo Trajano Gomes da Silva, James Ebdon, Joseph Okotto-Okotto, Frederick Ade, Oscar Mito, Peggy Wanza, Emmah Kwoba, Thumbi Mwangi, Weiyu Yu, Jim A. Wright

**Affiliations:** a School of Environment and Technology, University of Brighton, Cockcroft Building, Lewes Road, Brighton, BN2 4GJ, UK; b Victoria Institute for Research on Environment and Development (VIRED) International, P.O. Box 6423-40103, Off Nairobi Road, Rabour, Kisumu, Kenya; c Centre for Global Health Research, Kenya Medical Research Institute, P.O. Box 1578-40100, Kisumu, Kenya; d School of Geography and Environmental Science, University of Southampton, Building 44, Highfield, Southampton, SO17 1BJ, UK

**Keywords:** Water quality, Faecal indicator bacteria, Household water storage, Livestock, Health risks

## Abstract

**Background:**

Emerging evidence suggests close domestic proximity of livestock and humans may lead to microbiological contamination of hands, objects, food and water supplies within domestic environments, adversely impacting public health. However, evidence quantifying the relationship between livestock, domestic animals, humans and microbiological contamination of household stored water remains limited.

**Aim:**

This longitudinal study aimed to examine the relationship between domestic contact with livestock and domestic animals on microbiological contamination of household Point-of-Use (POU) stored drinking water in rural Kenya and assess the influence of choice of faecal indicator on such associations.

**Methodology:**

A survey was performed in 234 households in Siaya county, Kenya, to observe presence of livestock (cattle, goats, poultry) and domestic animals (cats, dogs) in household compounds, alongside other risk factors for contamination of POU stored drinking water such as sanitation, storage conditions and hygiene practices. Samples from water sources (e.g. piped, spring/wells, boreholes, surface and rainwater) and from POU storage containers were tested for *E. coli* and intestinal enterococci. Livestock-related risk factors for water contamination were examined through multinomial regression, controlling for confounders.

**Results:**

Rainwater was the main POU water source and was found to be highly susceptible to contamination. Multivariate analysis showed greater risk of gross (>100 CFU/100 mL) water contamination (with *E. coli*) for households where goats were observed, and/or where poultry roosted in proximity to stored household water (relative risk RR = 2.71; p = 0.001 and RR = 2.02; p = 0.012 respectively). Presence of a poultry coop was also associated with elevated intestinal enterococci densities (RR = 4.46; p = 0.001). Associations between contamination and livestock risk factors were thus similar for both bacteria groups, but *E. coli* counts declined more rapidly following collection from surface waters than enterococci counts (p = 0.024).

**Conclusion:**

The presence of livestock (particularly goats) and poultry within household compounds increases POU water contamination risk, suggesting the need for improved interventions to address cross-contamination within rural domestic settings. Within Siaya county, more effective community education is needed to raise awareness of POU water quality protection, particularly of rainwater.

## Highlights

• Poultry and goats are risk factors for household stored water contamination.
• Poultry are risk factors for both enterococci and *E. coli* contamination.
• Attenuation of enterococci in household stored water is lower than for *E. coli.*
• Residual free chlorine is mostly too low to prevent stored water recontamination.

## Graphical abstract

**Figure:**
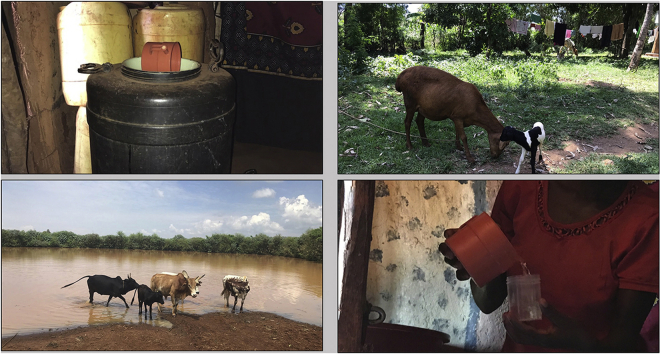

## 1 Introduction

UN Sustainable Development Goal (SDG 6) aims ‘to provide access to safe and affordable water, sanitation and hygiene for all by 2030’. The proportion of population using safely managed drinking water services increased from 61 to 71 percent between 2000 and 2017 (UN, 2020). According to the WHO/UNICEF, “improved” drinking water supply (used to monitor SDG 6) includes piped water, boreholes, protected springs, dug wells, and rainwater, while unprotected springs and dug wells, carts with small tanks, tanker trucks, and surface water are considered “unimproved” (Bartram et al., 2014; WHO/UNICEF, 2020). However, despite ever greater availability of improved water sources, microbial contamination of household stored water continues to present risks to human health in Low and Middle-Income Countries (LMIC). In 38% of studies included in a systematic review of drinking water quality in LMIC (Bain et al., 2014) over a quarter of samples from improved water sources contained faecal contamination. Wright et al. (2004) found that the bacteriological quality of drinking water frequently declines between source and point-of-use (POU) in LMIC because of risk factors such as lack of basic sanitation, use of uncovered storage vessels, storage vessel construction, and rurality. Several subsequent studies highlight faecal contamination of drinking-water from “improved” sources as a consequence of transportation, poor storage and handling practices (Classen and Bastable, 2003; Brick et al., 2004; Shaheed et al., 2014a; Rodrigues Peres et al., 2020). However, Shields et al. (2015) concluded that piped water in LMIC is less likely to be contaminated compared with other supply types at both source and POU. In summary, risks of microbial contamination of drinking water can arise during various stages en route from the water source to POU and are well documented in the extant literature.

WHO (2011) recommend the use of faecal indicator organisms (thermotolerant coliforms, *E. coli* and intestinal enterococci) to assess the health risks associated with drinking-water consumption. The presence of *E. coli* is associated with faecal contamination, and the WHO guidelines recommend the total absence of *E. coli* per 100 mL of drinking water. Presence of intestinal enterococci also indicates faecal contamination, but these microorganisms may persist longer in marine waters and be carried further than *E. coli* in the environment (WHO, 2011). Consequently, enterococci may indicate faecal contamination in water that might otherwise be missed. Although the WHO (2011) has not established a guideline value for enterococci, it states that its detection should lead to consideration of further action. Additionally, some research suggests that gastrointestinal disease is more strongly associated with the presence of enterococci than of *E. coli* (Barrel et al., 2000). Currently, the European Union's Drinking Water Directive (COUNCIL DIRECTIVE 98/83/EC, 1998) includes intestinal enterococci as a parameter for audit monitoring with a standard of 0 enterococci per 100 mL of water.

More recently, animal-related factors have been associated with moderate-to-severe human diarrhoeal infections, including household stored water as a transmission route (Zambrano et al., 2014; Conan et al., 2017). There are many enteric diseases attributable to contact with animals and their environments (Hale et al., 2012). For example, McDaniel et al. (2014) identified 45 pathogens capable of infecting humans and domestic cattle, with bacterial pathogens constituting the largest taxonomic group. Many zoonotic diarrhoeagenic enteric infections can be related to consumption of drinking-water contaminated with animal faeces. Daniels et al. (2015) detected *Cryptosporidium* and *Giardia* in Indian village tubewells and ponds. *Cryptosporidium* infection in rural Gambian children was linked to consumption of stored drinking-water and presence of cows and cats within household compounds (Hossain et al., 2019). Schriewer et al. (2015) used molecular Microbial Source Tracking (MST) techniques to validate human and animal faecal contamination of community water sources and stored drinking water in rural India. Hamzah et al. (2020) also applied MST techniques to assess post-supply contamination of drinking-water with ruminant faeces in 45 rural Kenyan households. Barnes et al. (2018) found household animal ownership and the presence of animal waste in household compounds were significantly associated with high enterococci counts in household drinking-water in peri-urban Kisumu, Kenya. In rural Bangladesh, Ercumen et al. (2017) found significantly higher *E. coli* levels in those households that contained animals. Thus, there is growing evidence linking domestic animals to stored water contamination in LMICs, but to date previous studies have either relied on a single faecal indicator, or analysed relatively small numbers of samples with molecular MST techniques.

This study aimed to add to this evidence-base by assessing the association between domestic contact with livestock and the microbial contamination of household POU stored water in Siaya County (Kenya), controlling for known risk factors for such contamination. As an auxiliary objective, the study also investigated whether apparent associations between POU water contamination and livestock are dependent on the choice of indicator bacteria group (namely *E. coli* versus intestinal enterococci).

## 2 Material and methods

### 2.1 Study site description

**Fig. 1:**
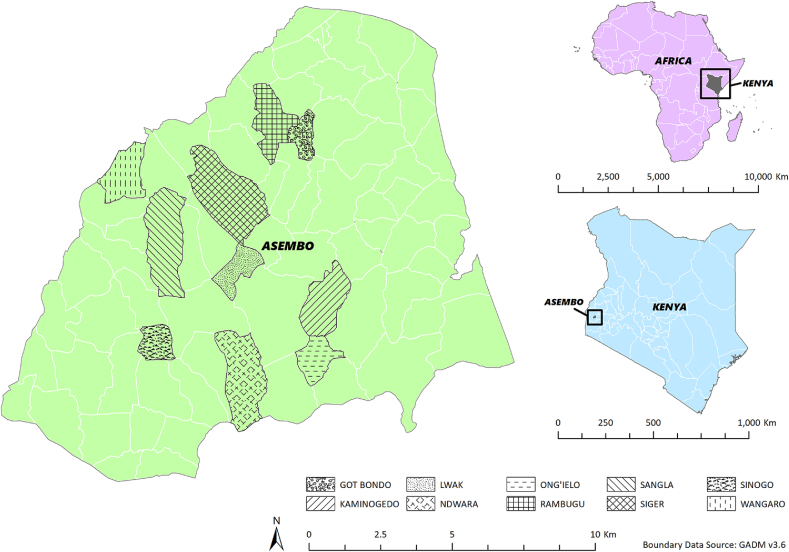
Location of the ten participating villages in Asembo, Siaya County, Kenya.

The study site was in Asembo, Siaya County, southwestern Kenya (Latitude: 0°3′46.2″S - 0°12′44.3″ South; Longitude: 34°16′51.6″E − 34°26′31.6″E; Altitude: approximately 1,200 m) and research took place in ten villages (Fig. 1) on the shores of Lake Victoria that also participate in an ongoing Population-Based Animal Syndrome Surveillance (PBASS) study (Odhiambo et al., 2012; Thumbi et al., 2015). Although some households use piped water, rainwater, hand-dug wells and boreholes, 29% of the County's households were using streams, rivers, dams and other surface waters as their main water source in 2011 (UNICEF, 2013). Most households used pit latrines in 2011, but 16% lacked any sanitation facilities. Smallholder agriculture is widespread, and within the PBASS study area, 55%, 19%, 41% and 88% own cattle, sheep, goats and poultry, respectively (Thumbi et al., 2015). These rural communities suffer concurrent high levels of poverty (Okwi et al., 2007) and high burden of infectious diseases (Feikin et al., 2011), including cryptosporidiosis (O'Reilly et al., 2012; Delahoy et al., 2018).

### 2.2 Study design

We conducted a longitudinal observational study of livestock-related risk factors for contamination of POU water with faecal indicator bacteria. Eligible study participants were adult members of households participating in the ongoing PBASS study, with children aged 6–59 months as the cohort at greatest risk of diarrhoeal disease. The sample size was powered to detect differential proportions of contaminated drinking-water using preliminary effect size estimates from Ghana (Wardrop et al., 2017), in the absence of evidence from Siaya. In Ghana, approximately 70% of water samples were contaminated in non-cattle keeping households, 90% were contaminated in cattle-keeping households, and the proportion of contamination variance in cattle-keeping households explained by other covariates was estimated at 0.3. Within the study population, 55% of households owned cattle (Thumbi et al., 2015). Based on these assumptions, a Type 1 error rate of 0.05, and a desired power of 0.9, a power calculation using the G* Power software indicated a required sample size of 196 households, which we rounded to 240 to allow for households declining to participate or dropping out of the study. Prior to recruitment, the study was registered with the International Standard Randomized Controlled Trial Number (ISRCTN) registry (Reference number: ISRCTN69058168).

## 3 Data collection

### 3.1 Household survey

Eligible households were randomly selected from lists of those participating in the PBASS study. After seeking informed consent, questionnaire interviews were conducted in the Dholuo language with adult respondents during an initial (Visit 1: 12th March to May 24, 2018) and follow-up sampling visit (Visit 2: November 20, 2018 to February 18, 2019). The initial and follow-up visits were intended to coincide with wet and dry seasons, respectively. To assess domestic contact with animals, interviewers observed the presence of livestock (e.g. cattle, goats, poultry), dogs and cats in the compound during interview and observed evidence of animal presence in the home (e.g. faeces; feathers; footprints). Compounds were designated as fenced areas surrounding homes. Interviewers also assessed whether drinking-water containers were accessible to animals and where poultry coops were observed, asked whether poultry were permanently confined in such coops. To measure other known risk factors for stored water contamination, water storage characteristics (e.g. whether containers were covered) were observed (Brick et al., 2004; Shaheed et al., 2014b; Thomas et al., 2015; Vannavong et al., 2018) and respondents asked about any water treatment or cleaning of storage vessels (Meierhofer et al., 2019). Interviewers also asked about sanitation facilities and handwashing behaviours, observing whether soap was available. To examine household socio-economic status as a potential risk factor for stored water contamination (Yang et al., 2013), we used a composite socioeconomic status score created using multiple correspondence analyses (MCA) of household assets, previously constructed for the PBASS study (Were et al., 2018). Data collection was performed using mobile smartphones containing the CommCare® data collection software (V.2.48.5).

### 3.2 Sample collection of source and household POU stored drinking water

After completing the questionnaire, the interviewer requested that the respondent fetch a sample of POU stored drinking-water (including any water stored separately for children), observing how the respondent collected water. Samples were also tested *in situ* for free residual chlorine using SenSafe Water Check test strips, capable of detecting 0, 0.05, 0.1, 0.2, 0.4, 0.6, 0.8, 1.2, 1.5, 2.0, 2.6, 4.0, and >6.0 mg/L. The method is approved by the US Environmental Protection Agency (ITS Method 99-003) (Part et al., 2007). On each day, a sample of stored drinking water was collected from storage containers of six to eight different households. Approximately 500 mL of water was collected by the respondent, using their own ladle or container, and decanted into sterile (autoclaved) polyethylene 1-L bottles (Fisher Scientific, UK). During both visits, a separate survey team also visited the sources of each household's stored drinking-water supply (as reported by households) and collected samples (where source water was still available). Depending on the source type, water was either decanted into sample bottles or bottles were dipped into the source (using sterile gloves and/or bottles attached to string).

### 3.3 Sample transport and processing

All sample bottles were kept in a cooled container (Approx. 4 °C) and transported within 4 h to the Kenya Medical Research Institute (KEMRI) laboratories in Kisian, Kisumu. Samples were either processed immediately or kept in the fridge (4 °C) and processed within 24 h. Microbiological water quality was assessed via faecal indicator bacteria (FIB), namely *E. coli* and intestinal enterococci.

Enumeration of FIB followed ISO standard methods (ISO 9308-1:2014 for *Escherichia coli* and total coliforms, and ISO 7899-2:2000 for Intestinal enterococci) and was performed using membrane filtration (Anon, 2000, Anon, 2017). As pilot studies revealed that some 10 and 100 mL water samples contained organisms which were Too Numerous To Count (TNTC), it was decided to additionally filter 0.1 and 1 mL volumes, to ensure countable, evenly spread colonies were obtained. All water samples were tested in quadruplicate for each FIB investigated, comprising of four volumes (0.1, 1, 10 and 100 mL) filtered through a 0.45 μm pore-size cellulose nitrate filter (Thermo Scientific) using a vacuum filtration unit (Fisher®). Approximately 10 mL of quarter-strength Ringer's (QSR) solution was added as this diluent aids enumeration by ensuring that FIB colonies are evenly distributed over the filter, and not clumped together.

Total coliforms and *E. coli* detection: The filters were placed onto coliform chromogenic agar (CCE) agar (Difco®) in Ø 55 mm petri plates (Fisher®). Plates were then incubated upside down for 24 ± 2 h at 37.0 ± 0.5 °C. Colonies that showed shades of dark-blue to violet were counted as *E. coli*, while those that appeared pink to red-coloured were recorded as presumptive coliforms (total coliforms) that were not *E. coli* (ISO 9308-1:2014) (Anon, 2017).

Intestinal enterococci detection: The filters were placed onto Slanetz and Bartley agar (Oxoid®) in Ø 55 mm petri plates (Fisher®). Plates were then incubated upside down for 48 ± 2 h at 37.0 ± 0.5 °C. Colonies (raised) coloured red, maroon or pink were counted as presumptive intestinal enterococci (ISO 7899/2:2000) (Anon, 2000).

From the four volumes (0.1, 1, 10 and 100 mL) filtered for each sample, the plate used for enumeration was based on the highest countable volume (100 mL) that was not TNTC. All FIB results were expressed as colony-forming units (CFU) per 100 mL.

### 3.4 Precipitation estimation

Given reported associations between water supply contamination and rainfall (Egwari and Aboaba, 2002), daily rainfall was also measured through extraction from the Climate Hazard group InfraRed Precipitation with Station (CHIRPS) dataset (Funk et al., 2015). We acquired the latest version of the 0.05° (~5 km) gridded CHIRPS dataset (CHIRPS v2.0), produced by blending *in-situ* station observations with satellite-based precipitation estimates. The 5 km × 5 km CHIRPS grid cells were overlaid on study village boundaries using ArcGIS 10.4.1 (ESRI, Redlands, CA, USA). For each fieldwork date between March 12, 2018 and February 18, 2019, average daily rainfall over the entire study area was determined as the area-weighted average of overlapping grid cell values.

### 3.5 Statistical analysis

Statistical analysis was undertaken in IBM SPSS v25.0 and Stata v16 (StataCorp, 2019).Only *E. coli* and intestinal enterococci were used in the main statistical analyses, since they are WHO's chosen indicator microorganisms for assessing microbiological drinking water quality. Water source types with small sample sizes were grouped prior to analysis as follows: ‘Piped’ (Piped water into dwelling; piped water into yard; public standpipe; water kiosk), ‘Well/Spring’ (protected well; unprotected spring), ‘Surface Water’ (river or stream; dam, pond or lake). Boreholes and harvested rainwater were included in analysis without grouping. Kruskal-Wallis statistical analyses were performed to test for significant differences in *E. coli* and enterococci levels between the five source type groups (‘Piped’, ‘Well/Spring’, ‘Rainwater’ ‘Surface Water’ and ‘Borehole’).

To visualise bacterial contamination data, bacteria counts were logged, first replacing left-censored values with 0.5 and right-censored with values immediately above the upper limit of detection (202,000 for total coliforms, 11,400 CFU/100 mL for *E. coli* and 20,200 CFU/100 ml for intestinal enterococci). Logged POU FIB counts and the change in counts between source and POU were then plotted as strip charts for surface and piped water supplies, the only source types with sufficient data for such visualization. A Wilcoxon signed rank test for matched pairs was used to test for differences in attenuation for *E. coli* versus enterococci.

Given the statistical difficulties of handling values outside the limits of detection, POU samples were categorised as having low (<10 CFU/100 ml), medium (10–99 CFU/100 ml) or high (≥100 CFU/100 ml) faecal contamination. Following examination of cross-tabulations of FIB levels versus risk factors, univariate multinomial regression was used to examine the relationship between each risk factor and contamination. Robust regression in Stata v16 (StataCorp, 2019) was used to account for clustering in samples drawn from the same household in successive visits. A multivariate, multinomial regression model was then developed, following Hosmer and Lemeshow (2013). Covariates with P values greater than 0.25 were removed from this model whose exclusion did not substantially change the coefficients of remaining covariates or substantially affect the likelihood ratio of the fitted model. The reported source of each POU water supply was retained in this model, given its importance for interpretation.

## 4 Results

### 4.1 POU stored water sampling flow

**Fig. 2:**
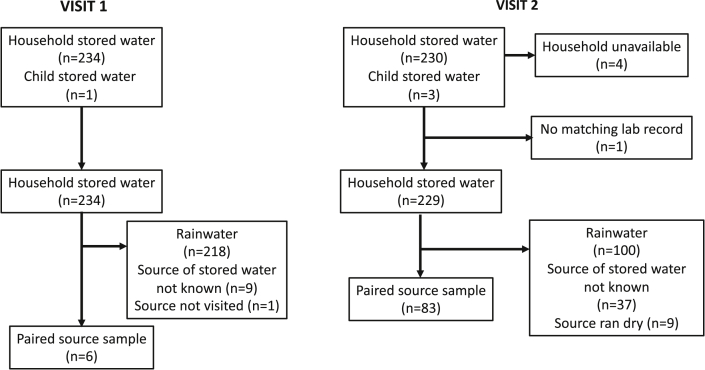
POU stored water sampling flow for household visit 1 (12th March - May 24, 2018) and visit 2 (Nov 20, 2018–Feb 18, 2019).

Fig. 2 below illustrates sample characteristics from the two visits, including the number of stored drinking water samples that could be paired with the original source. 234 households were recruited to the study, but four of these were unavailable during the second visit (and were omitted from subsequent analyses). There were very few instances of water stored exclusively for child use; therefore, such instances were not included in data analysis.

Most stored drinking-water was harvested rainwater, corresponding to 93.1% and 43.6% of samples from visit 1 and 2, respectively. As average daily rainfall fell between visit 1 (7.749 mm/day) and visit 2 (2.354 mm/day), use of non-rainwater sources increased significantly (by 22.6%, 10.0% and 3.8% in piped, surface and spring/well water, respectively), as did the range of source types used (six in visit 1 versus nine in visit 2). It was not possible to collect ‘paired’ source samples to compare with household water in 218 households during visit 1 (and 100 households during visit 2), because either rainwater had been harvested directly into storage vessels, or there was no longer harvested rainwater available. Across both visits, respondents were unsure of the exact source of stored water on 46 occasions, which also prevented ‘paired’ source samples being collected. Nine sources had run dry during visit 2, so could not be sampled. Thirty-nine (47%) of 83 water source samples were collected within 2 days of POU sampling, but 34 (41%) were collected on dates more than a week apart. Thirty-eight (46%) of source samples were collected prior to POU samples, 33 (40%) afterwards, and 12 (14%) on the same day.

### 4.2 Household survey

Most household survey respondents were the household head's spouse (73.5%), the person responsible for domestic water management (81.6%), and female (83.2%). To store drinking-water, 60.3% of households used a small container (≤20 L); 31.3% used a large container (>20 L and ≤ 100 L) and 7.5% used a water tank (>100 L). 98.1% of these containers/tanks were located inside the household. 52.8% self-reported that they did not perform any water treatment. Of those that did, 6.7% boiled their water; 18.5% used chlorination; 5.0% added coagulant; 27.2% strained water through a piece of cloth and just 0.6% filtered water. 74.8% of households had a pit latrine with slab and 5.8% had a ventilated improved pit latrine (VIP), both constituting improved sanitation. However, 16.4% of households practiced open defecation (OD) and 4.5% had a pit latrine without slab, constituting unimproved sanitation. Interviewers observed poultry in 90.5% of household compounds; cattle in 53.7%; dogs in 48.5%; cats in 45.9%; and goats in 35.1%. 65.7% of drinking-water vessels were accessible to domesticated or wild animals.

### 4.3 Microbial contamination of stored drinking water

Summary statistics for FIB levels according to the source of stored water can be found in supplementary material (SM1). Table 1 displays median levels (CFU/100 mL) of FIB (*E. coli* and intestinal enterococci) by source of POU stored water.

**Table 1:** Overall median levels (CFU/100 mL) of *E. coli* and intestinal enterococci by POU stored drinking water, according to water source type for two combined household visits (1 & 2).

Source type (n)	Water source (n)	Median (CFU/100 mL)	
		*E. coli*	Intestinal enterococci
Piped (71)	Piped water into dwelling (1)	23	51
	Piped water into yard (33)	39	51
	Public standpipe (34)	15	34
	Water Kiosk (3)	4	4
Rainwater (324)	Rainwater (324)	11	100
Spring/well (12)	Protected well (9)	11	87
	Unprotected spring (3)	29	96
Surface water (31)	River or stream (2)	86	566
	Dam, pond or lake (29)	30	58
Borehole (3)	Borehole or tubewell (3)	<1	1
Unknown (23)	Unknown (23)	29	12

N = number of observations.

**Fig. 3:**
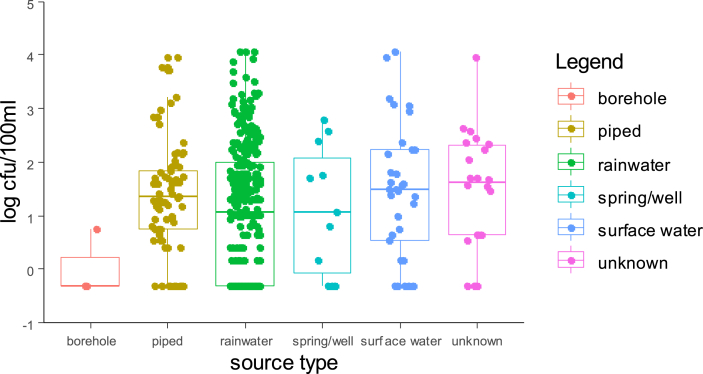
Box-plots with median levels (CFU/100 mL) of *E. coli* by source type of POU stored drinking water for two successive household visits (bar: median; boxes: 25th and 75th centiles; whiskers: 1.5 times inter-quartile range).

**Fig. 4:**
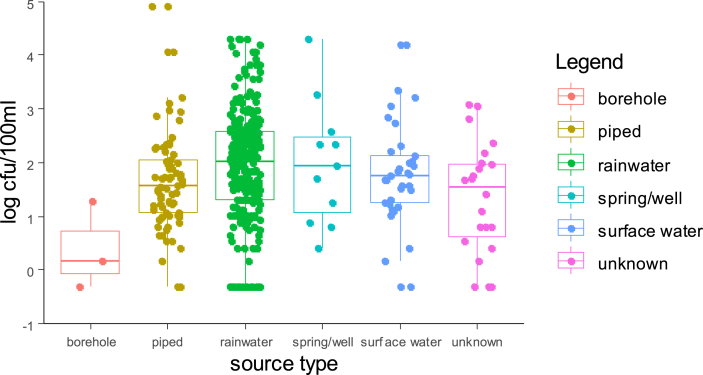
Box-plots with median levels (CFU/100 mL) of intestinal enterococci by source type of POU stored drinking water for two successive household visits (bar: median; boxes: 25th and 75th centiles; whiskers: 1.5 times inter-quartile range).

Overall detected levels of intestinal enterococci (Md = 69 CFU/100 mL) were statistically (p < 0.001) above those of *E. coli* (Md = 17.50 CFU/100 mL) for all source types with the exception of water kiosks. Fig. 3, Fig. 4 present jittered box-plots displaying FIB levels (*E. coli* and intestinal enterococci) by water source type (Piped; Rainwater; Spring/well; Surface water; Borehole; Unknown).

Fig. 3, Fig. 4 demonstrate that harvested rainwater is the preferred source type and that FIB levels varies greatly within each source type. Furthermore, harvested rainwater displayed the highest median level for intestinal enterococci, which was significantly greater than piped and borehole median values (p = 0.06 and 0.019, respectively) (See Supplementary material SM2). The local population perceived rainwater as “blessed”, and fewer households reported treating rainwater than surface water (51.9% versus 84%). 24% of piped water underwent treatment.

**Fig. 5:**
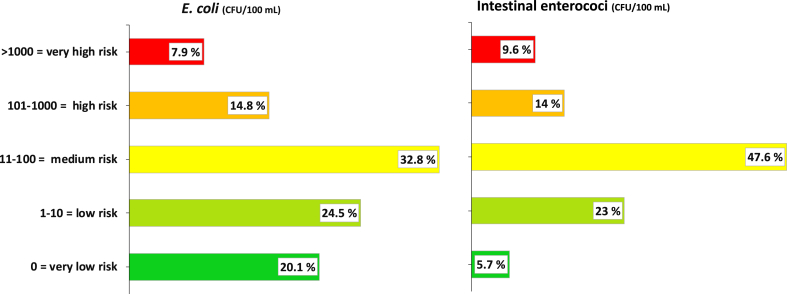
Percentage of POU stored drinking water samples (n = 229) according to FIB health risk categories (Lloyd and Bartram, 1991) during visit 2.

Supplemental material SM3 shows percentages of samples by FIB health risks categories (Lloyd and Bartram, 1991) during two successive household visits. During pilot studies, some water samples generated Too Numerous To Count (TNTC) results for 10 mL volumes and almost all for 100 mL volumes, so the laboratory team decided to filter only 0.1, 1 mL and 10 ml volumes for visit 1 samples. Subsequently, data from visit 1 suggested that bacterial contamination levels were lower than in pilot fieldwork, so 100 ml samples were additionally processed in visit 2, thereby enabling assessment of *E. coli* compliance with the WHO guideline value (‘0’ or not detectable in 100 ml). Fig. 5 below displays percentages of samples in each FIB health risks category (Lloyd and Bartram, 1991) for visit 2 only, given that no 100 ml volumes were processed in visit 1.

Only 20% (46/229) and 6% (13/229) of samples from visit 2 were negative for the presence of *E. coli* and enterococci in 100 mL of POU water, respectively and met WHO (2011) and (EU (1998) guidelines. High faecal contamination levels were observed in POU water, with 33% of samples for *E. coli* and 48% for intestinal enterococci constituting a ‘moderate risk’ to population health, with 23% for *E. coli* and intestinal enterococci a ‘high’ or ‘very high’ risk (Lloyd and Bartram, 1991).

Strip tests indicated six of 50 samples (12%) reportedly treated using home chlorination contained a free chlorine residual of ≥ 0.2 ppm, whilst 12 (24%) contained no detectable free chlorine residual. Two of 59 (3.4%) piped water samples contained a free chlorine residual of ≥ 0.2 ppm, with 23 (39.0%) containing no detectable free chlorine residual.

### 4.4 Analysis of risk factors for POU water

Table 2, Table 3 respectively, display number and percentages of samples where *E. coli* and intestinal enterococci contamination levels in POU stored drinking water are cross-tabulated against risk factors associated with animal contact. Supplementary Material Table SM4 contains similar information, but includes all risk factors controlled for in regression analysis of POU water contamination.

**Table 2:** Number (and percentage) of POU stored drinking water samples in each *E. coli* contamination category, cross-tabulated against contamination risk factors associated with animal contact.

Risk factors	Number (%) of POU samples classed as			Total
	Low contamination (<10 CFU/100 mL)	Medium contamination (10–99 CFU/100 mL)	High contamination (≥ 100 CFU/100 mL)	
	*E. coli*	*E. coli*	*E. coli*	
***Animals observed in household compound:***				
Goats	52 (31.9%)	58 (35.6%)	53 (32.5%)	163
Cattle	100 (40.2%)	87 (34.9%)	62 (24.9%)	249
Dogs	86 (38.4%)	72 (32.1%)	66 (29.5%)	224
Cats	88 (41.3%)	66 (31.0%)	59 (27.7%)	213
Poultry	166 (39.5%)	146 (34.8%)	108 (25.7%)	420
Poultry (confined in coop)	130 (37.6%)	119 (34.4%)	97 (28.0%)	346
Poultry spend night by stored water	83 (34.2%)	89 (36.6%)	71 (29.2%)	243
Signs of livestock inside home	142 (38.7%)	126 (34.3%)	99 (27.0%)	367
***Water storage container:***				
Water container accessible to animals	108 (35.4%)	102 (33.4%)	95 (31.2%)	305
**All samples**	**184 (39.7%)**	**157 (33.9%)**	**122 (26.4%)**	**463**

**Table 3:** Number and percentage of POU stored drinking water samples in each intestinal enterococci contamination category, cross-tabulated against contamination risk factors associated with animal contact.

Risk factors	Number (%) of POU samples classed as			Total
	Low contamination (<10 CFU/100 mL)	Medium contamination (10–99 CFU/100 mL)	High contamination (≥ 100 CFU/100 mL)	
	Intestinal enterococci	Intestinal enterococci	Intestinal enterococci	
***Animals observed in household compound:***				
Goats	29 (17.8%)	59 (36.2%)	75 (46.0%)	163
Cattle	52 (20.9%)	86 (34.5%)	111 (44.6%)	249
Dogs	45 (20.1%)	72 (32.1%)	107 (47.8%)	224
Cats	33 (15.5%)	75 (35.2%)	105 (49.3%)	213
Poultry	75 (17.9%)	152 (36.2%)	193 (46.0%)	420
Poultry (confined in coop)	52 (15.0%)	114 (33.0%)	180 (52.0%)	346
Poultry spend night by stored water	37 (15.2%)	97 (39.9%)	109 (44.9%)	243
Signs of livestock inside home	65 (17.7%)	124 (33.8%)	178 (48.5%)	367
***Water storage container:***				
Water container accessible to animals	48 (15.7%)	110 (36.1%)	147 (48.2%)	305
**All samples**	**86 (18.6%)**	**172 (37.2%)**	**205 (44.3%**	**463**

In contrast to *E. coli*, samples from households where coops were used to confine poultry had the highest percentage of samples containing >100 CFU/100 mL of intestinal enterococci (52.0%). In all such cases, poultry were allowed to roam free for some time.

In unadjusted multinomial regression analysis, observations of domestic animal contacts were associated with medium contamination with *E. coli* only when poultry spent the night in proximity to stored water (relative risk [RR] = 1.59; p = 0.044) (Table 4, Table 5), and this remained significant in models adjusted for confounders (such as improved sanitation, reported home chlorination, …) (relative risk [RR] = 2.23; p = 0.003). However, the probability of water being grossly contaminated (>100 CFU/100 mL) with *E. coli* was significantly higher for households where goats were observed (relative risk [RR] = 1.95; p = 0.01) and where poultry reportedly spent the night near stored water (RR = 1.69; p = 0.026). Both risk factors remained significant in models adjusted for confounders (RR = 2.71; p = 0.001 and RR = 2.02; p = 0.012 respectively). For the unadjusted model of *E. coli*, the risk of water being grossly contaminated was significantly higher for households in which storage containers were accessible to animals (RR = 2.48; p = 0.001) (Table 4), but not in the adjusted model. Of the confounders controlled for (Tables SM5 and SM6), the presence of soap within the household, improved sanitation, reported home chlorination, and reported cleaning of storage container lids all significantly reduced the risk of high *E. coli* contamination in unadjusted models. Absence of a lid/cover on water storage vessels increased the risk of high *E. coli* contamination (SM5).

**Table 4:** Adjusted and unadjusted relative risk ratios for contact with domestic animals versus household POU stored drinking water contamination with *E. coli*, as derived from multinomial regression.

Risk factor	Unadj. bivariate regression		Adj. multivariate regression	
	Relative risk ratio (95% ci)	P value	Relative risk ratio (95% ci)	P value
**Medium contamination (10–99 CFU/100 ml)**				
***Livestock observed in household compound:***				
Goats	1.49 (0.91–2.44)	0.116	1.46 (0.80–2.65)	0.213
Cattle	1.04 (0.68–1.61)	0.846		
Dogs	0.97 (0.63–1.48)	0.872		
Cats	0.79 (0.50–1.26)	0.321		
Poultry	1.44 (0.65–3.19)	0.369		
Poultry (confined in coop)	1.19 (0.71–2.01)	0.506		
Poultry spend night by stored water	1.59 (1.01–2.51)	0.044*	2.23 (1.30–3.83)	0.003**
Signs of livestock inside home	1.20 (0.70–2.06)	0.501		
Water storage container accessible to animals	1.31 (0.83–2.06)	0.253		
**High contamination (** > = **100 CFU/100 ml)**				
Goats	1.95 (1.17–3.24)	0.010**	2.71 (1.51–4.87)	0.001**
Cattle	0.87 (0.54–1.41)	0.565		
Dogs	1.34 (0.83–2.18)	0.234		
Cats	1.02 (0.63–1.66)	0.931		
Poultry	0.84 (0.39–1.81)	0.650		
Poultry (confined in coop)	1.69 (0.92–3.10)	0.092		
Poultry spend night by stored water	1.69 (1.07–2.69)	0.026*	2.02 (1.17–3.50)	0.012*
Signs of livestock inside home	1.27 (0.71–2.27)	0.414		
Water storage container accessible to animals	2.48 (1.42–4.31)	0.001**		

* = significant at the 0.05 level/** = significant at the 0.01 level.

**Table 5:** Adjusted and unadjusted relative risk ratios for contact with domestic animals versus household POU stored drinking water contamination with intestinal enterococci, as derived from multinomial regression.

Risk factor	Unadj. bivariate regression		Adj. multivariate regression	
	Relative risk ratio (95% ci)	P value	Relative risk ratio (95% ci)	P value
**Medium contamination (10–99 CFU/100 ml)**				
***Livestock observed in household compound:***				
Goats	1.03 (0.59–1.78)	0.926		
Cattle	0.65 (0.38–1.11)	0.116		
Dogs	0.66 (0.39–1.11)	0.117		
Cats	1.24 (0.72–2.14)	0.436		
Poultry	1.11 (0.50–2.48)	0.79		
Poultry (confined in coop)	1.18 (0.66–2.12)	0.569	1.51 (0.67–3.38)	0.320
Poultry spend night by stored water	1.71 (0.99–2.96)	0.053		
Signs of livestock in home	0.83 (0.46–1.51)	0.551		
Water storage container accessible to animals	1.40 (0.85–2.32)	0.186		
**High contamination (** > = **100 CFU/100 ml)**				
Goats	1.13 (0.68–1.89)	0.629		
Cattle	0.77 (0.47–1.27)	0.309		
Dogs	0.99 (0.61–1.63)	0.983		
Cats	1.69 (1.01–2.79)	0.042*		
Poultry	2.36 (1.02–5.45)	0.044*		
Poultry (confined in coop)	5.19 (2.64–10.21)	< 0.001**	4.46 (1.80–11.07)	0.001**
Poultry spend night by stored water	1.50 (0.90–2.52)	0.121		
Signs of livestock in home	2.13 (1.14–3.98)	0.018*		
Water storage container accessible to animals	2.01 (1.21–3.33)	0.007*		

* = significant at the 0.05 level/** = significant at the 0.01 level.

For the unadjusted model of intestinal enterococci, the risk of water being grossly contaminated was significantly greater for households where either cats (RR = 1.69; p = 0.042) and/or poultry (RR = 2.36; p = 0.044) were observed, and/or when storage containers were accessible to animals (RR = 2.01; p = 0.007). These variables were no longer significant in a preliminary multivariate model, however. Also, the presence of a coop to confine poultry was associated with greater risk of high contamination (RR = 5.19; p < 0.001). Furthermore, this risk factor remained significant in the adjusted model (RR = 4.46; p = 0.001) (Table 5). Of the confounders controlled for (SM6), presence of soap in the household, improved sanitation, and reported cleaning of storage container lids all significantly reduced high intestinal enterococci contamination risk in unadjusted models. Reported home chlorination reduced the risk of high intestinal enterococci contamination in adjusted models. In contrast, when dirt was observed on water storage containers, the risk of high intestinal enterococci contamination increased (SM6).

### 4.5 Microbial contamination of source versus POU water quality

**Fig. 6:**
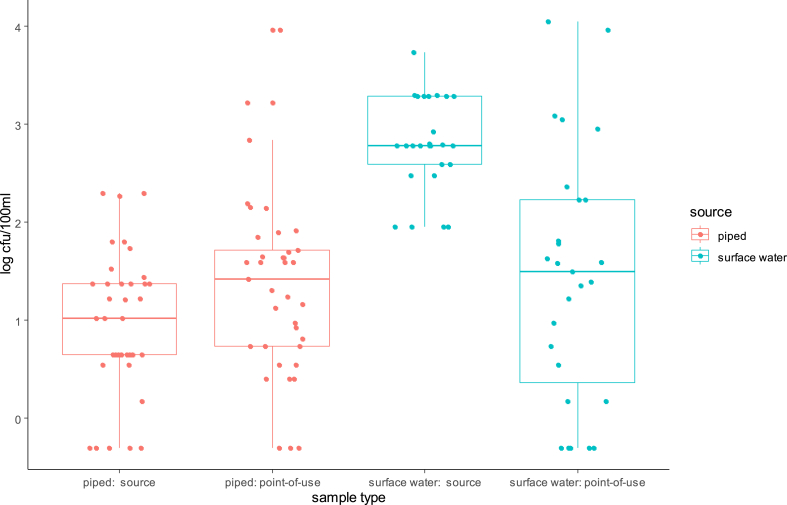
Microbial contamination with *E. coli* of source water versus POU stored drinking water samples in 37 households using piped or kiosk water and 27 households using surface waters.

**Fig. 7:**
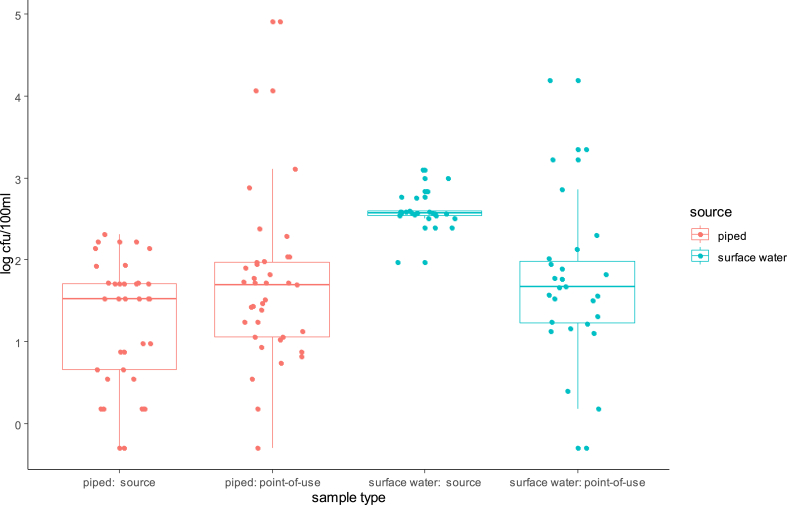
Microbial contamination with intestinal enterococci of source water versus POU stored drinking water samples in 37 households using piped or kiosk water and 27 households using surface waters.

Paired sample analysis (Fig. 6, Fig. 7) suggested that surface water sources contained significantly higher levels of both FIB than piped water (p < 0.001), with this difference being particularly large for *E. coli*. However, POU samples originating from piped versus surface water sources had similar median levels of FIB. Since initial *E. coli* levels in surface source waters were greater than enterococci levels in such waters, *E. coli* counts thus declined more rapidly following water collection than enterococci counts, as shown by a Wilcoxon signed rank test (p = 0.024). As mentioned previously, 84% of surface water sources underwent treatment when compared to 24% of piped sources.

## 5 Discussion

After controlling for other known risk factors such as inadequate sanitation, our findings suggests that the presence of poultry in household compounds (as indicated by poultry coops) is associated with high POU water contamination. The presence of goats was also associated with POU water contamination with *E. coli*. This study thus meets calls for greater evidence on domestic exposure to contamination from livestock based on direct observations of livestock presence, as opposed to reported livestock ownership (Penakalapati et al., 2017). Harvested rooftop rainwater was the major source of POU drinking water during this research. Despite being classified as an improved water source by the JMP (WHO/UNICEF, 2020), rainwater was heavily contaminated by faecal bacteria. This observation concurs with Shaheed et al. (2014a) who suggested that improved water sources are not always safe, and more efforts should be placed into water testing and POU treatment. Our finding that poultry-keeping increased drinking-water contamination risk is supported by findings elsewhere. Avian faecal contamination (from wild birds) and pathogen presence in rooftop harvested rainwater (Ahmed et al., 2016, 2011; Chidamba and Korsten, 2015; Hamilton et al., 2019) is also well documented. Furthermore, young children's hand contact with poultry faeces in the household compound (Marquis et al., 1990) was identified as a risk factor for diarrhoea, environmental enteric disorder (EED) and respiratory infections (Headey et al., 2017; Headey and Hirvonen, 2016). Our identification of goat-keeping as a particular risk is somewhat supported by Schriewer et al. (2015), who found the livestock ruminant *Bacteroidales* MST molecular marker present in 96% of stored drinking-water from 24 Indian villages. Harris et al. (2016) also found evidence via MST molecular techniques of faecal contamination from ruminants (with goats observed in 10% of households, cattle in 3%) on children's hands and on household floors in Dhaka, Bangladesh. In Kakamega county (Kenya), Hamzah et al. (2020) found the ruminant *Bacteroides* MST marker present in stored drinking-water within 64% of participating households but not in source waters, suggesting post-collection ruminant faecal contamination. However, only 4% of the households visited in their study owned goats compared to 47% owning cattle. During the study design phase of our project, we hypothesized that cattle ownership was a likely risk factor for POU water contamination, based on the fact that cattle ownership has been shown to increase *E. coli* contamination of POU water in Ghana and Bangladesh (Wardrop et al., 2018). However, we found no evidence of such risk from cattle presence in the compound in multinomial regression models presented here. Other more general evidence linking domestic animals, water contamination and related infections includes Barnes et al. (2018), who found domestic animal ownership in urban Kisumu (Kenya) increased risk of enterococci contamination of POU drinking-water. Hossain et al. (2019) found increased risk of *Cryptosporidium* infections with consumption of stored drinking water where animals (cats and cattle) were present within household compounds.

It is known that *E. coli* outnumber intestinal enterococci in human faeces (Zubrzycki and Spaulding, 1961). In domestic animals, Harris et al. (2016) found *E. coli* concentrations to be 0.7, 3.0 and 2.0 Log_10_ higher than intestinal enterococci in chicken, cow and goat faeces, respectively. In our study, median enterococci levels in surface water samples were lower than *E. coli* levels (Fig. 6, Fig. 7.), but higher in the household POU stored water (Table 1). Attenuation of enterococci levels between surface sources and POU was thus less than for *E. coli,* suggesting that it is likely to be a more conservative and reliable indicator of risk to health. These findings differ from a previous study that simultaneously examined enterococci and *E. coli* in both source water and POU water sources from Ecuadorian villages (Levy et al., 2008), which found very similar median levels of the two FIB and similar patterns of attenuation and recontamination. Studies report variable persistence of enterococci in freshwater, depending on seasonal ultra-violet exposure and bacterial sources (Staley et al., 2014). In freshwater, *E. coli* decay rates were significantly lower than those of enterococci (Anderson et al., 2005). Furthermore, according Boehm and Sassoubre (2014), intestinal enterococci is much more persistent in marine/brackish waters when compared to *E. coli*.

Despite the apparent greater contamination, and/or persistence of enterococci in POU stored water environment, the association of water contamination with livestock risk factors is broadly consistent for both FIB groups. This is similar to other studies of non-livestock based risk factors for POU contamination (Levy et al., 2008) involving both FIB groups. For both *E. coli* and enterococci, poultry-keeping was associated with higher bacteria levels, whilst for both FIB groups, water containers being accessible to animals was a risk factor for contamination in unadjusted but not adjusted models. However, interestingly the presence of goats was a risk factor for *E. coli* contamination only.

The adjusted effect of other known risk factors for POU contamination (SM5 and SM6) was generally consistent with findings of previous studies, suggesting plausible bacterial contamination patterns. For example, high cumulative rainfall preceding sampling increased risk of stored water contamination with both FIB in our study, and a Rwandan study (Kirby et al., 2016) similarly found extreme rainfall immediately prior to sampling increased thermotolerant coliform levels in household stored water. In-house chlorination significantly reduced the odds of high enterococci contamination (>100 CFU/100 mL), as it did for both *E. coli* and enterococci contamination of POU water in Ecuador (Levy et al., 2008). However, in contrast to surface water users in Ghana (Wright et al., 2016), low-income households in our study were no more exposed to contaminated stored water than wealthier households, though this may in part reflect a lack of socio-economic differentiation within our the study area.

Following systematic review recommendations (Shields et al., 2015), we measured free chlorine alongside FIB in POU water samples. For piped samples, chlorine residual was frequently not detectable, suggesting that chlorination procedures were inadequate for preventing subsequent (re)contamination. Furthermore, where households reported chlorinating stored drinking-water, there was often no detectable chlorine residual, reflecting other studies that have found limited effectiveness and consistent use of household-level water treatment (Clasen, 2015; Rosa et al., 2014). Since drinking-water access is typically measured at household rather than individual level, analogously to food security measurement (Pinstrup-Andersen, 2009), there is potential for exposure misclassification where individuals within the same household access water in different ways. In this population, it was rare that water for young children was stored separately/differently from water used by other household members, suggesting that such misclassification seldom occurs.

### 5.1 Limitations

Given that *Cryptosporidium* has been implicated in child diarrhoea in Siaya County (Delahoy et al., 2018; O'Reilly et al., 2012), caution is needed in interpreting FIB changes between source and POU. Since people and cattle, a host for *C. parvum*, often share surface waters in the study area and oocysts typically persist for longer than FIB in freshwater, the similar FIB levels in POU samples from surface waters versus other source types may mask a public health risk from surface water consumption. Whilst it would be preferable to test POU water for *Cryptosporidium* as the principal pathogen of concern rather than FIB, the very large sample volumes needed (typically>20 L) for this (Muchiri et al., 2009) present both logistical and ethical obstacles to such a study design. Source samples were sometimes collected over a week before or after POU samples, so given temporal variation in source quality (Levy et al., 2008), this inhibited our ability to measure bacterial recontamination or die-off. Apparent associations in our data could be due to unmeasured confounding risk factors, such as rainwater contamination from wild bird faeces. As noted in previous reviews (Wright et al., 2004), recall bias could have affected reporting of household water treatment and the reported source of stored drinking-water. However, most survey respondents were also the household members responsible for water management within the home. Whilst all selected households agreed to participate in the study, the PBASS study that provided our sampling framework could have been subject to some limited selection bias during its recruitment.

### 5.2 Interpretation

Given the risk factors for POU water contamination identified here, there are several candidate interventions that could potentially reduce such contamination. As poultry-keeping and presence of goats were identified as risk factors for contamination, interventions for corralling goats and poultry in the home could merit investigation (Zambrano et al., 2014). However, our findings suggests sustained rather than intermittent use of coops for confining poultry would be needed to reduce contamination. Well-established candidate interventions include safe water storage and household water treatment via chlorination, flocculation/chlorination or home filtration (Clasen, 2015). Although only 5% of households reported treating water by flocculation, given high turbidity of surface waters in this area, use of combined flocculation/chlorination could be more effective than chlorination alone (WHO, 2019). However, Meschke et al. (2009) hypothesized that human factors (including improper storage and chlorine dosing) may reduce effectiveness of POU chlorine disinfection. Since many of our reportedly chlorinated POU samples had no detectable free chlorine, further education is needed concerning appropriate practice, dosage and barriers remain to its effective use. In a low-income community in Dhaka (Bangladesh) Pickering et al. (2015), reported that chlorine tablet usage fell by 50% after behavioural promotion visits ended, suggesting intensive promotion is necessary for sustained uptake, as argued by others (Geremew et al., 2019). Alternatively, POU drinking water filtration has previously been used successfully in western Kenya (Morris et al., 2018), where a randomized controlled trial found 71% of filtered water samples in compliance with WHO guidelines and where households using ceramic filters reported less diarrhoea/fewer health facility visits for diarrhoea. Fagerli et al. (2020) assessed the efficacy and health impact of hollow fibre ultrafilters for water treatment in rural Kenyan households and found reduced odds of *E. coli* contamination in stored water for intervention households, but no difference in the odds of reported infant diarrhoea.

However, ceramic filters are not efficient at removing viruses (Van der Laan et al., 2014) and protect against bacteria and protozoa only (WHO, 2019), whereas chlorine disinfection protects against bacteria and viruses only (though not protozoa). In summary, ideally household water treatment interventions in Siaya should follow a multi-barrier approach, adopting multiple treatment technologies to achieve comprehensive protection.

In keeping with another recent study in western Kenya (Thomson et al., 2019), rainwater harvesting was widely practiced and a preferred water source among our study's participants. However, such water was seldom treated and was regarded as safe, so given similar FIB levels detected in POU samples originating from rainwater and surface water, the importance of safe handling and treatment of drinking-water from all sources should be emphasised to the population. Various treatment technologies could be used to further reduce microbial contamination of harvested rainwater, such as first flush diversion, or filtration (e.g. slow sand filtration or gravel boxes), UV solar disinfection (SODIS), solar pasteurization (SOPAS), chemical disinfection (chlorination) or a combination of these technologies (Hamilton et al., 2019).

The population studied has some distinctive characteristics that may reduce generalisability of findings. For instance, many households in our study consumed untreated surface waters, yet only 12.8% of rural Sub-Saharan African households did so in 2017 (UNICEF and WHO, 2017), making this population somewhat unusual. A particularly strong trust between the village communities and researchers has developed through multiple longitudinal studies, which could have reduced risk of inaccurate answers to recall-based questions in the household survey, but also potentially have led to questionnaire fatigue from participation in multiple studies (Egleston et al., 2011).

## 6 Conclusion

After controlling for other known risk factors, there is evidence that poultry-keeping in household compounds increases the risk of POU water being highly contaminated. This finding holds true when contamination is measured with both enterococci and *E. coli*, despite differing source water contamination levels and subsequent attenuation following storage in the home for these two bacterial groups. Additionally, presence of goats in the household compound was associated with high enterococci contamination of POU water. Both *E. coli* and intestinal enterococci appear to provide similar information regarding the likely risks associated with POU drinking water and source water, but intestinal enterococci appears to be a more conservative indicator, due to its extended persistence within such settings. The study highlighted the need for awareness-raising among the local population about managing contamination of harvested rainwater. Candidate interventions to manage POU water safety should be based on the multibarrier approach and include household treatment technologies, and steps to safely separate of livestock and humans within the home.

## Funding

This research is a contribution to the OneHealthWater project (http://www.onehealthwater.org/), which received funding from the 10.13039/501100000265UK Medical Research Council/10.13039/501100002992Department for International Development via a 10.13039/100016270Global Challenges Research Fund foundation grant (Ref.: MR/P024920/1). The study sponsors had no role in the subsequent execution of the study. This UK funded award is part of the EDCTP2 programme supported by the European Union.

## Ethics approval

Ethical approval for the study was obtained from the Faculty of Social and Human Sciences, University of Southampton (references: 31554 and 48834; approval dates: February 12, 2018 and April 23, 2019) and the Kenya Medical Research Institute (reference: KEMRI/SERU/CGHR/091/3493, approval date: October 17, 2017).

## Availability of data and material

The datasets on water samples collected and household questionnaires used in the study are available from the corresponding author on reasonable request and are available in the UK Data Archive repository at http://doi.org/10.5255/UKDA-SN-854302. The datasets on precipitation used and analysed in this study are available from the CHIRPS website at http://chg.geog.ucsb.edu/data/chirps/.

## Authors’ contributions

Diogo Trajano Gomes da Silva (DTGS), Joseph Okotto-Okotto, Jim Wright (JW) and Thumbi Mwangi designed the study. Emmah Kwoba and Peggy Wanza undertook data capture and archiving. Oscar Mito and Frederick Ade undertook laboratory work. Weiyu Yu (WY) undertook spatial data capture and archiving. DTGS, JW and WY undertook visualization of the published work. DTGS and JW analysed data from the study. James Ebdon (JE) supervised the study. DTGS, JE and JW drafted the manuscript with the support of the other authors.

## Declaration of competing interest

The authors declare no conflict of interest.
