# Supplementary material for: A longitudinal study of the association between domestic contact with livestock and contamination of household point-of-use stored drinking water in rural Siaya County (Kenya)
Source: Int J Hyg Environ Health. 2020 Sep;230:113602. doi: 10.1016/j.ijheh.2020.113602 (PMC7607227; doi:10.1016/j.ijheh.2020.113602)
Supplement: Multimedia component 1 [file mmc1.docx]

SM1_Summaries statistics of FIB for all POU water sources
