# Supplementary figures and images for: A longitudinal study of the association between domestic contact with livestock and contamination of household point-of-use stored drinking water in rural Siaya County (Kenya)
Source: Int J Hyg Environ Health. 2020 Sep;230:113602. doi: 10.1016/j.ijheh.2020.113602 (PMC7607227; doi:10.1016/j.ijheh.2020.113602)

SM2_Kruskall Wallis statistical analyses for POU water sources types.


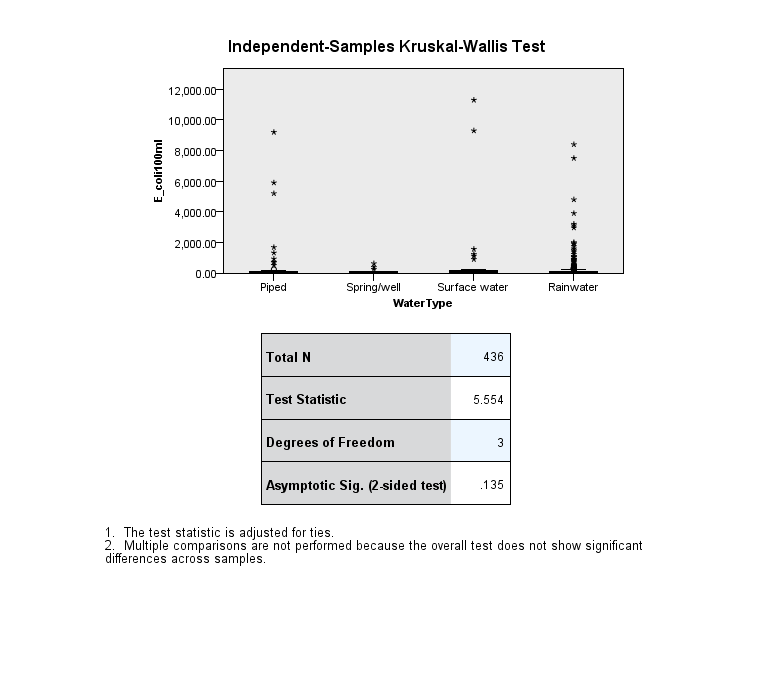


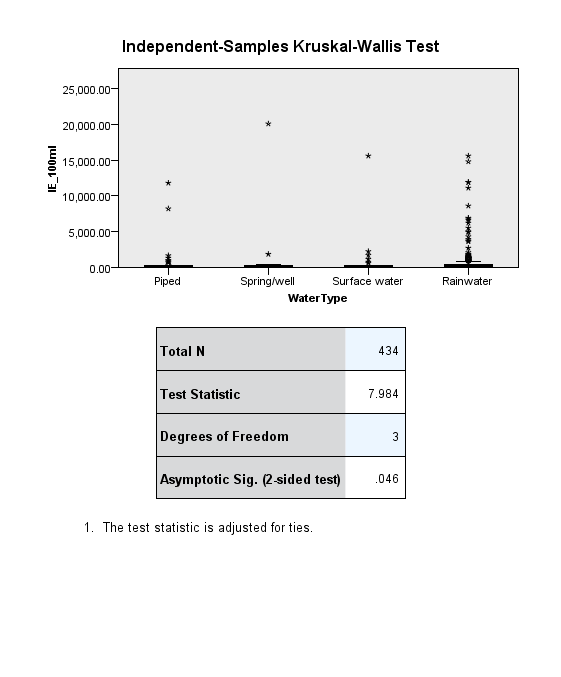


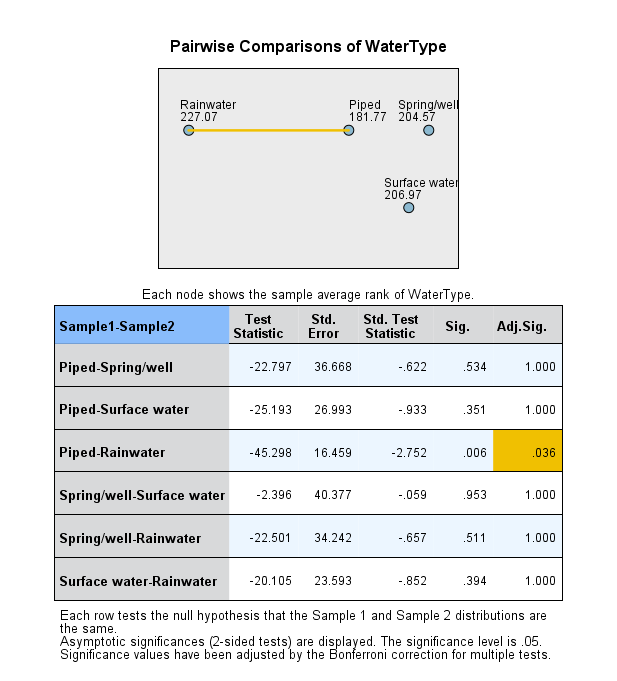

Supplement: Multimedia component 2 [file mmc2.docx]
