# Supplementary material for: A longitudinal study of the association between domestic contact with livestock and contamination of household point-of-use stored drinking water in rural Siaya County (Kenya)
Source: Int J Hyg Environ Health. 2020 Sep;230:113602. doi: 10.1016/j.ijheh.2020.113602 (PMC7607227; doi:10.1016/j.ijheh.2020.113602)
Supplement: Multimedia component 3 [file mmc3.docx]

**SM3.** Percentages of POU water samples according to FIB health risks categories (Lloyd and Bartram 1991) during two successive sampling visits.

| **FIB levels (CFU/ 100 mL)** | **FIB (%)** | | | | | |
| --- | --- | --- | --- | --- | --- | --- |
|  | **Visit 1** | | **Visit 2** | | **Overall** | |
|  | **E coli** | **Intestinal enterococci** | **E coli** | **Intestinal enterococci** | **E coli** | **Intestinal enterococci** |
| **0 = very low risk** | 35.5* | 10.9* | 20.1 | 5.7 | 27.8 | 8.3 |
| **1-10 = low risk** | 13 | 4.4 | 24.5 | 23.1 | 18.7 | 13.8 |
| **11-100 = medium risk** | 26 | 22.7 | 32.8 | 47.6 | 29.3 | 35.2 |
| **101-1000 = high risk** | 20.3 | 51.5 | 14.8 | 14 | 17.6 | 32.8 |
| **>1000 = very high risk** | 5.2 | 10.5 | 7.9 | 9.6 | 6.5 | 10 |

* Detected in 10 mL sample volumes
