# Supplementary material for: A longitudinal study of the association between domestic contact with livestock and contamination of household point-of-use stored drinking water in rural Siaya County (Kenya)
Source: Int J Hyg Environ Health. 2020 Sep;230:113602. doi: 10.1016/j.ijheh.2020.113602 (PMC7607227; doi:10.1016/j.ijheh.2020.113602)
Supplement: Multimedia component 4 [file mmc4.docx]

### SM4. Number and ﻿percentage of POU water samples in *E. coli* and intestinal enterococci contamination categories, cross-tabulated against contamination risk factors

| **Risk factors** | **Number (%) of POU samples classed as** | | | | | | **Total** |
| --- | --- | --- | --- | --- | --- | --- | --- |
|  | **Low contamination**  **(< 10 CFU /100 mL)** | | **Medium contamination**  **(10-99 CFU / 100mL)** | | **High contamination**  **(>= 100 CFU/100mL)** | |  |
|  | ***E. coli*** | **Intestinal enterococci** | ***E. coli*** | **Intestinal enterococci** | ***E. coli*** | **Intestinal enterococci** |  |
| ***Reported source of stored water:*** |  |  |  |  |  |  |  |
| - Piped / kiosk / borehole | 29 (39.2%) | 16 (21.6%) | 30 (40.5%) | 38 (51.4%) | 15 (20.3%) | 20 (27.0%) | 74 |
| - Rainwater / well spring | 136 (40.7%) | 58 (17.4%) | 110 (32.9%) | 106 (31.7%) | 88 (26.4%) | 170 (50.9%) | 334 |
| - Surface water | 11 (33.3%) | 3 (9.1%) | 11 (33.3%) | 20 (60.6%) | 11 (33.3%) | 10 (30.3%) | 33 |
| ***Sanitation & hygiene:*** |  |  |  |  |  |  |  |
| Soap observed in household | 91 (45.3%) | 42 (20.9%) | 65 (32.3%) | 85 (42.3%) | 45 (22.4%) | 74 (37.8%) | 201 |
| No sanitation | 24 (31.6%) | 7 (9.2%) | 29 (38.2%) | 26 (34.2%) | 23 (30.3%) | 43 (56.6%) | 76 |
| Improved sanitation* | 155 (41.6%) | 77 (20.6%) | 128 (34.3%) | 146 (39.1%) | 90 (24.1%) | 150 (40.2%) | 373 |
| ***Animals observed in household compound:*** | | | | | | | |
| - Goats | 52 (31.9%) | 29 (17.8%) | 58 (35.6%) | 59 (36.2%) | 53 (32.5%) | 75 (46.0%) | 163 |
| - Cattle | 100 (40.2%) | 52 (20.9%) | 87 (34.9%) | 86 (34.5%) | 62 (24.9%) | 111 (44.6%) | 249 |
| - Dogs | 86 (38.4%) | 45 (20.1%) | 72 (32.1%) | 72 (32.1%) | 66 (29.5%) | 107 (47.8%) | 224 |
| - Cats | 88 (41.3%) | 33 (15.5%) | 66 (31.0%) | 75 (35.2%) | 59 (27.7%) | 105 (49.3%) | 213 |
| - Poultry | 166 (39.5%) | 75 (17.9%) | 146 (34.8%) | 152 (36.2%) | 108 (25.7%) | 193 (46.0%) | 420 |
| - Poultry (confined in coop) | 130 (37.6%) | 52 (15.0%) | 119 (34.4%) | 114 (33.0%) | 97 (28.0%) | 180 (52.0%) | 346 |
| - Poultry spend night by stored water | 83 (34.2%) | 37 (15.2%) | 89 (36.6%) | 97 (39.9%) | 71 (29.2%) | 109 (44.9%) | 243 |
| - Signs of livestock inside home | 142 (38.7%) | 65 (17.7%) | 126 (34.3%) | 124 (33.8%) | 99 (27.0%) | 178 (48.5%) | 367 |
| ***Water storage and handling:*** | | | | | | | |
| Did not wash hands before fetching water | 158 (39.8%) | 73 (18.4%) | 131 (33.0%) | 146 (36.8%) | 108 (27.2%) | 178 (44.8%) | 397 |
| Hand made contact with water when dipping | 13 (43.3%) | 3 (10.0%) | 6 (20.0%) | 10 (33.3%) | 11 (36.7%) | 17 (56.7%) | 30 |
| No lid /cover on water container | 18 (27.7%) | 9 (13.9%) | 25 (38.5%) | 22 (33.9%) | 22 (33.9%) | 34 (52.3%) | 65 |
| Water stored below waist height | 154 (38.1%) | 73 (18.1%) | 138 (34.2%) | 147 (36.4%) | 112 (27.7%) | 184 (45.5%) | 404 |
| Water container dirty | 93 (36.9%) | 32 (12.7%) | 86 (34.1%) | 84 (33.3%) | 73 (29.0%) | 136 (54.0%) | 252 |
| Water container accessible to animals | 108 (35.4%) | 48 (15.7%) | 102 (33.4%) | 110 (36.1%) | 95 (31.2%) | 147 (48.2%) | 305 |
| ***Reported cleaning of storage container:*** | | | | | | | |
| - Lid cleaned | 154 (42.4%) | 75 (20.7%) | 120 (33.1%) | 140 (38.6%) | 89 (24.5%) | 148 (40.8%) | 363 |
| - Inside cleaned | 172 (40.4%) | 80 (18.8%) | 141 (33.1%) | 160 (37.6%) | 113 (26.5%) | 186 (43.7%) | 426 |
| - With soap/detergent | 119 (41.3%) | 54 (18.8%) | 95 (33.0%) | 109 (37.9%) | 74 (25.7%) | 125 (43.4%) | 288 |
| - Today/yesterday | 38 (35.2%) | 19 (17.6%) | 42 (38.9%) | 37 (34.3%) | 28 (25.9%) | 52 (48.2%) | 108 |
| - In last week | 118 (42.0%) | 56 (19.9%) | 83 (29.5%) | 109 (38.8%) | 80 (28.5%) | 116 (41.3%) | 281 |
| - Longer than a week | 17 (48.6%) | 7 (20.0%) | 13 (37.1%) | 11 (31.4%) | 5 (14.3%) | 17 (48.6%) | 35 |
| ***Reported water treatment:*** | | | | | | | |
| - Boiled | 17 (54.8%) | 5 (16.1%) | 7 (22.6%) | 14 (45.2%) | 7 (22.6%) | 12 (38.7%) | 31 |
| - Strained | 43 (34.1%) | 17 (13.5%) | 47 (37.3%) | 45 (35.7%) | 36 (28.6%) | 64 (50.8%) | 126 |
| - Chlorinated | 40 (46.5%) | 22 (25.6%) | 32 (37.2%) | 30 (34.9%) | 14 (16.3%) | 34 (39.5%) | 86 |
| Free residual chlorine (<0.2mg/L) | 172 (40.4%) | 79 (18.5%) | 146 (34.3%) | 159 (37.3%) | 108 (25.4%) | 188 (44.1%) | 426 |
| ***Wealth quintile:*** | | | | | | | |
| - Poorest | 18 (30.5%) | 12 (20.3%) | 23 (39.0%) | 19 (32.2%) | 18 (30.5%) | 28 (47.5%) | 59 |
| - Poor | 49 (43.4%) | 20 (17.7%) | 33 (29.2%) | 49 (43.4%) | 31 (27.4%) | 44 (38.9%) | 113 |
| - Middle | 29 (37.2%) | 13 (16.7%) | 29 (37.2%) | 30 (38.5%) | 20 (25.6%) | 35 (44.9%) | 78 |
| - Rich | 46 (41.4%) | 18 (16.2%) | 37 (33.3%) | 39 (35.1%) | 28 (25.2%) | 54 (48.7%) | 111 |
| - Richest | 42 (41.2%) | 23 (22.6%) | 35 (34.3%) | 35 (34.3%) | 25 (24.5%) | 44 (43.1%) | 102 |
| ***Rainfall in preceding 10 days >=50mm*** | 74 (35.4%) | 29 (13.9%) | 69 (33.0%) | 52 (24.9%) | 66 (31.6%) | 128 (61.2%) | 209 |
| **Total** | 184 (39.7%) | 86 (18.6%) | 157 (33.9%) | 172 (37.2%) | 122 (26.4%) | 205 (44.3%) | 463 |

* = VIP latrine or pit with slab
