# Supplementary material for: A longitudinal study of the association between domestic contact with livestock and contamination of household point-of-use stored drinking water in rural Siaya County (Kenya)
Source: Int J Hyg Environ Health. 2020 Sep;230:113602. doi: 10.1016/j.ijheh.2020.113602 (PMC7607227; doi:10.1016/j.ijheh.2020.113602)
Supplement: Multimedia component 5 [file mmc5.docx]

## **SM5.** Bivariate and multivariate multinomial regression coefficients of risk factors for POU water contamination with *E. coli*

| **Risk factor** | **Unadj. bivariate regression** | | **Adj. multivariate regression** | |  |
| --- | --- | --- | --- | --- | --- |
|  | **Relative risk ratio (95% ci)** | **P value** | **Relative risk ratio (95% ci)** | **P value** |  |
| **Medium contamination (10-99 CFU/100ml)** | | | | |  |
| ***Reported source of stored water (reference: rainwater, well or spring):*** | | | | | |
| Piped / kiosk / borehole | 1.28 (0.70 to 2.34) | 0.424 | 1.26 (0.60 to 2.68) | 0.541 |  |
| Surface water | 1.24 (0.51 to 3.01) | 0.640 | 1.09 (0.39 to 3.05) | 0.868 |  |
| ***Sanitation & hygiene:*** | | | | |  |
| Soap observed in household | 0.72 (0.46 to 1.14) | 0.159 |  |  |  |
| No sanitation | 1.51 (0.85 to 2.69) | 0.162 |  |  |  |
| Improved sanitation (VIP latrine or pit with slab) | 0.83 (0.47 to 1.44) | 0.499 |  |  |  |
| ***Animals observed in household compound:*** | | | | | |
| Goats | 1.49 (0.91 to 2.44) | 0.116 | 1.46 (0.80 to 2.65) | 0.213 |  |
| Cattle | 1.04 (0.68 to 1.61) | 0.846 |  |  |  |
| Dogs | 0.97 (0.63 to 1.48) | 0.872 |  |  |  |
| Cats | 0.79 (0.50 to 1.26) | 0.321 |  |  |  |
| Poultry | 1.44 (0.65 to 3.19) | 0.369 |  |  |  |
| Poultry (confined in coop) | 1.19 (0.71 to 2.01) | 0.506 |  |  |  |
| Poultry spend night by stored water | 1.59 (1.01 to 2.51) | 0.044* | 2.23 (1.30 to 3.83) | 0.003** |  |
| Signs of livestock inside home | 1.20 (0.70 to 2.06) | 0.501 |  |  |  |
| ***Water storage and handling:*** | | | | |  |
| Free residual chlorine <0.2mg/L | 0.90 (0.39 to 2.09) | 0.806 |  |  |  |
| Did not wash hands before fetching water | 0.83 (0.45 to 1.51) | 0.541 |  |  |  |
| Hand made contact with water when dipping | 0.55 (0.21 to 1.39) | 0.205 | 0.22 (0.06 to 0.73) | 0.014 |  |
| No lid /cover on water container | 1.75 (0.93 to 3.27) | 0.081 |  |  |  |
| Water stored below waist height | 1.41 (0.76 to 2.64) | 0.277 |  |  |  |
| Water container dirty | 1.19 (0.75 to 1.87) | 0.465 |  |  |  |
| Water container accessible to animals | 1.31 (0.83 to 2.06) | 0.253 |  |  |  |
| ***Reported cleaning of storage container:*** | | | | | |
| Lid cleaned | 0.63 (0.37 to 1.08) | 0.092 | 0.83 (0.42 to 1.66) | 0.607 |  |
| Inside cleaned | 0.61 (0.29 to 1.33) | 0.215 |  |  |  |
| With soap / detergent | 0.89 (0.53 to 1.47) | 0.638 |  |  |  |
| ***Container last cleaned (reference: today or yesterday)*** | | | | | |
| - In last week | 0.64 (0.38 to 1.08) | 0.091 | 0.54 (0.31 to 0.95) | 0.031 |  |
| - Longer than a week | 0.69 (0.30 to 1.61) | 0.393 | 0.67 (0.27 to 1.68) | 0.394 |  |
| **Reported water treatment (reference: no or any other form of treatment):** | | | | |  |
| - Boiled | 0.46 (0.19 to 1.13) | 0.089 |  |  |  |
| - Chlorinated | 0.92 (0.55 to 1.55) | 0.759 | 1.05 (0.59 to 1.86) | 0.865 |  |
| - Strained | 1.40 (0.86 to 2.29) | 0.177 |  |  |  |
| ***Wealth quintile (reference: poorest)*** | | | | | |
| Poor | 0.53 (0.24 to 1.14) | 0.105 |  |  |  |
| Middle | 0.78 (0.35 to 1.76) | 0.553 |  |  |  |
| Rich | 0.63 (0.29 to 1.36) | 0.238 |  |  |  |
| Richest | 0.65 (0.30 to 1.43) | 0.287 |  |  |  |
| Rainfall in preceding 10 days *>=50mm* | 1.005 (0.996 to 1.013) | 0.288 | 1.010 (0.999 to 1.020) | 0.064 |  |
| **High contamination (>=100 CFU /100ml)** | | | | | |
| ***Reported source of stored water (reference: rainwater, well or spring):*** | | | | | |
| Piped / kiosk / borehole | 0.80 (0.40 to 1.59) | 0.525 | 1.16 (0.52 to 2.60) | 0.719 |  |
| Surface water | 1.55 (0.68 to 3.50) | 0.297 | 1.30 (0.51 to 3.28) | 0.585 |  |
| ***Sanitation & hygiene:*** | | | | |  |
| Soap observed in household | 0.60 (0.37 to 0.97) | 0.035* |  |  |  |
| No sanitation | 1.55 (0.79 to 3.04) | 0.203 |  |  |  |
| Improved sanitation (VIP latrine or pit with slab) | 0.53 (0.29 to 0.95) | 0.034* |  |  |  |
| ***Animals observed in household compound:*** | | | | | |
| Goats | 1.95 (1.17 to 3.24) | 0.010** | 2.71 (1.51 to 4.87) | 0.001** |  |
| Cattle | 0.87 (0.54 to 1.41) | 0.565 |  |  |  |
| Dogs | 1.34 (0.83 to 2.18) | 0.234 |  |  |  |
| Cats | 1.02 (0.63 to 1.66) | 0.931 |  |  |  |
| Poultry | 0.84 (0.39 to 1.81) | 0.650 |  |  |  |
| Poultry (confined in coop) | 1.69 (0.92 to 3.10) | 0.092 |  |  |  |
| Poultry spend night by stored water | 1.69 (1.07 to 2.69) | 0.026* | 2.02 (1.17 to 3.50) | 0.012* |  |
| Signs of livestock inside home | 1.27 (0.71 to 2.27) | 0.414 |  |  |  |
| ***Water storage and handling:*** | | | | |  |
| Free residual chlorine <0.2mg/L | 0.52 (0.22 to 1.20) | 0.125 |  |  |  |
| Did not wash hands before fetching water | 0.83 (0.45 to 1.51) | 0.541 |  |  |  |
| Hand made contact with water when dipping | 1.34 (0.58 to 3.13) | 0.497 | 1.21 (0.43 to 3.43) | 0.716 |  |
| No lid /cover on water container | 2.03 (1.00 to 4.10) | 0.049* |  |  |  |
| Water stored below waist height | 2.18 (0.98 to 4.84) | 0.055 |  |  |  |
| Water container dirty | 1.46 (0.88 to 2.42) | 0.145 |  |  |  |
| Water container accessible to animals | 2.48 (1.42 to 4.31) | 0.001** |  |  |  |
| ***Reported cleaning of storage container:*** | | | | | |
| Lid cleaned | 0.53 (0.29 to 0.94) | 0.030* | 0.44 (0.21 to 0.94) | 0.034* |  |
| Inside cleaned | 0.88 (0.37 to 2.08) | 0.764 |  |  |  |
| With soap / detergent | 0.81 (0.47 to 1.39) | 0.452 |  |  |  |
| **Container last cleaned (reference: today or yesterday):** | | | | | |
| In last week | 0.92 (0.53 to 1.58) | 0.763 | 0.76 (0.43 to 1.36) | 0.352 |  |
| Longer than a week | 0.40 (0.14 to 1.13) | 0.085 | 0.30 (0.10 to 0.93) | 0.037 |  |
| **Reported water treatment (reference: no or any other form of treatment):** | | | | | |
| Boiled | 0.60 (0.25 to 1.44) | 0.251 |  |  |  |
| Chlorinated | 0.47 (0.24 to 0.90) | 0.022* | 0.51 (0.25 to 1.04) | 0.063 |  |
| Strained | 1.37 (0.82 to 2.31) | 0.233 |  |  |  |
| ***Wealth quintile (reference: poorest)*** | | | | | |
| Poor | 0.63 (0.29 to 1.38) | 0.250 |  |  |  |
| Middle | 0.69 (0.28 to 1.68) | 0.414 |  |  |  |
| Rich | 0.61 (0.27 to 1.36) | 0.225 |  |  |  |
| Richest | 0.60 (0.25 to 1.41) | 0.240 |  |  |  |
| Rainfall in preceding 10 days (>=50mm) | 1.012 (1.003 to 1.021) | 0.008** | 1.012 (1.001 to 1.023) | 0.032* |  |

* = significant at the 0.05 level / ** = significant at the 0.01 level
