# Supplementary material for: A longitudinal study of the association between domestic contact with livestock and contamination of household point-of-use stored drinking water in rural Siaya County (Kenya)
Source: Int J Hyg Environ Health. 2020 Sep;230:113602. doi: 10.1016/j.ijheh.2020.113602 (PMC7607227; doi:10.1016/j.ijheh.2020.113602)
Supplement: Multimedia component 6 [file mmc6.docx]

## **SM6**.Bivariate and multivariate multinomial regression coefficients of risk factors for POU water contamination with Intestinal enterococci

| **Risk factor** | **Unadj. bivariate regression** |  | **Adj. multivariate regression** |  |
| --- | --- | --- | --- | --- |
|  | **Relative risk ratio (95% ci)** | **P value** | **Relative risk ratio (95% ci)** | **P value** |
| **Medium contamination (10-99 CFU/100ml)** | | | | |
| ***Reported source of stored water (reference: rainwater, well or spring):*** | | | | |
| Piped / kiosk / borehole | 1.30 (0.67 to 2.51) | 0.435 | 1.53 (0.71 to 3.32) | 0.281 |
| Surface water | 3.65 (1.04 to 12.8) | 0.044* | 3.67 (0.94 to 14.2) | 0.061 |
| ***Sanitation & hygiene:*** | | | | |
| Soap observed in household | 1.02 (0.61 to 1.72) | 0.930 |  |  |
| No sanitation | 2.01 (0.81 to 5.00) | 0.133 | 1.70 (0.63 to 4.59) | 0.291 |
| Improved sanitation (VIP latrine or pit with slab) | 0.66 (0.28 to 1.52) | 0.325 |  |  |
| ***Animals observed in household compound*** | | | | |
| Goats | 1.03 (0.59 to 1.78) | 0.926 |  |  |
| Cattle | 0.65 (0.38 to 1.11) | 0.116 |  |  |
| Dogs | 0.66 (0.39 to 1.11) | 0.117 |  |  |
| Cats | 1.24 (0.72 to 2.14) | 0.436 |  |  |
| Poultry | 1.11 (0.50 to 2.48) | 0.79 |  |  |
| Poultry (confined in coop) | 1.18 (0.66 to 2.12) | 0.569 | 1.51 (0.67 to 3.38) | 0.320 |
| Poultry spend night by stored water | 1.71 (0.99 to 2.96) | 0.053 |  |  |
| Signs of livestock in home | 0.83 (0.46 to 1.51) | 0.551 |  |  |
| ***Water storage and handling:*** |  |  |  |  |
| Free residual chlorine <0.2mg/L | 1.12 (0.43 to 2.89) | 0.825 |  |  |
| Did not wash hands before fetching water | 1.00 (0.46 to 2.16) | 1.00 |  |  |
| Hand made contact with water when dipping |  |  |  |  |
| No lid /cover on water container | 1.25 (0.57 to 2.77) | 0.574 |  |  |
| Water stored below waist height | 1.05 (0.52 to 2.12) | 0.898 |  |  |
| Water container dirty | 1.61 (0.96 to 2.69) | 0.069 | 1.55 (0.79 to 3.05) | 0.206 |
| Water container accessible to animals | 1.40 (0.85 to 2.32) | 0.186 |  |  |
| ***Reported cleaning of storage container:*** | | | | |
| Lid cleaned | 0.64 (0.31 to 1.34) | 0.239 | 0.63 (0.26 to 1.50) | 0.294 |
| Inside cleaned | 1.00 (0.36 to 2.79) | 1.00 |  |  |
| With soap/detergent | 1.11 (0.62 to 2.00) | 0.721 |  |  |
| ***Container last cleaned (reference: today or yesterday)*** | | | | |
| - In last week | 1.00 (0.52 to 1.92) | 0.999 |  |  |
| - Longer than a week | 0.81 (0.27 to 2.43) | 0.703 |  |  |
| **Reported water treatment (reference: no or any other form of treatment):** | | | | |
| - Boiled | 1.44 (0.55 to 3.73) | 0.458 |  |  |
| - Chlorinated | 0.61 (0.34 to 1.10) | 0.102 | 0.47 (0.24 to 0.90) | 0.022 |
| - Strained | 1.44 (0.77 to 2.70) | 0.259 |  |  |
| ***Wealth quintile (reference: poorest)*** | | | | |
| Poor | 1.55 (0.62 to 3.85) | 0.348 |  |  |
| Middle | 1.46 (0.53 to 4.06) | 0.471 |  |  |
| Rich | 1.37 (0.54 to 3.49) | 0.512 |  |  |
| Richest | 0.96 (0.37 to 2.47) | 0.934 |  |  |
| Rainfall in preceding 10 days (mm) | 0.99 (0.98 to 1.00) | 0.249 | 0.998 (0.984 to 1.011) | 0.742 |
| **High contamination (>=100 CFU/100ml)** | | | | |
| ***Reported source of stored water (reference: rainwater, well or spring):*** | | | | |
| Piped / kiosk / borehole | 0.43 (0.21 to 0.89) | 0.022* | 1.04 (0.43 to 2.52) | 0.928 |
| Surface water | 1.14 (0.30 to 4.30) | 0.850 | 2.55 (0.59 to 11.0) | 0.210 |
| ***Sanitation & hygiene:*** |  |  |  |  |
| Soap observed in household | 0.59 (0.36 to 0.99) | 0.044* |  |  |
| No sanitation | 3.00 (1.35 to 6.63) | 0.007** | 2.17 (0.85 to 5.59) | 0.107 |
| Improved sanitation (VIP latrine or pit with slab) | 0.32 (0.15 to 0.66) | 0.002** |  |  |
| ***Animals observed in household compound*** | | | | |
| Goats | 1.13 (0.68 to 1.89) | 0.629 |  |  |
| Cattle | 0.77 (0.47 to 1.27) | 0.309 |  |  |
| Dogs | 0.99 (0.61 to 1.63) | 0.983 |  |  |
| Cats | 1.69 (1.01 to 2.79) | 0.042* |  |  |
| Poultry | 2.36 (1.02 to 5.45) | 0.044* |  |  |
| Poultry (confined in coop) | 5.19 (2.64 to 10.2) | <0.001** | 4.46 (1.8 to 11.07) | 0.001** |
| Poultry spend night by stored water | 1.50 (0.90 to 2.52) | 0.121 |  |  |
| Signs of livestock in home | 2.13 (1.14 to 3.98) | 0.018* |  |  |
| ***Water storage and handling:*** |  |  |  |  |
| Free residual chlorine <0.2mg/L | 1.02 (0.39 to 2.69) | 0.972 |  |  |
| Did not wash hands before fetching water | 1.17 (0.55 to 2.49) | 0.675 |  |  |
| Hand made contact with water when dipping |  |  |  |  |
| No lid /cover on water container | 1.70 (0.77 to 3.77) | 0.191 |  |  |
| Water stored below waist height | 1.56 (0.80 to 3.04) | 0.191 |  |  |
| Water container dirty | 3.33 (2.03 to 5.46) | <0.001** | 1.63 (0.85 to 3.11) | 0.142 |
| Water container accessible to animals | 2.01 (1.21 to 3.33) | 0.007** |  |  |
| ***Reported cleaning of storage container:*** | | | | |
| Lid cleaned | 0.38 (0.19 to 0.77) | 0.008** | 0.30 (0.12 to 0.71) | 0.006** |
| Inside cleaned | 0.73 (0.28 to 1.94) | 0.533 |  |  |
| With soap/detergent | 1.04 (0.62 to 1.75) | 0.878 |  |  |
| **Container last cleaned (reference: today or yesterday)** | | | | |
| In last week | 0.76 (0.40 to 1.44) | 0.395 |  |  |
| Longer than a week | 0.89 (0.31 to 2.54) | 0.824 |  |  |
| **Reported water treatment (reference: no or any other form of treatment):** | | | | |
| Boiled | 1.01 (0.34 to 2.98) | 0.990 |  |  |
| Chlorinated | 0.58 (0.31 to 1.07) | 0.082 | 0.42 (0.21 to 0.82) | 0.011* |
| Strained | 1.84 (0.99 to 3.42) | 0.052 |  |  |
| ***Wealth quintile (reference: poorest)*** | | | | |
| Poor | 0.94 (0.43 to 2.04) | 0.882 |  |  |
| Middle | 1.15 (0.46 to 2.85) | 0.757 |  |  |
| Rich | 1.29 (0.59 to 2.80) | 0.528 |  |  |
| Richest | 0.82 (0.37 to 1.83) | 0.628 |  |  |
| Rainfall in preceding 10 days (mm) (>=50mm) | 1.02 (1.01 to 1.03) | <0.001** | 1.017 (1.003 to 1.03) | 0.011* |

* = significant at the 0.05 level / ** = significant at the 0.01 level
